# The Development of Ambiguity Processing Is Explained by an Inverted U-Shaped Curve

**DOI:** 10.3390/bs14090826

**Published:** 2024-09-16

**Authors:** Anna Manelis, Rachel Miceli, Skye Satz, Stephen J. Suss, Hang Hu, Amelia Versace

**Affiliations:** 1 Department of Psychiatry, University of Pittsburgh, Pittsburgh, PA 15213, USAssuss007@fiu.edu (S.J.S.); versacea@upmc.edu (A.V.); 2 Department of Radiology, Magnetic Resonance Research Center, University of Pittsburgh Medical Center, University of Pittsburgh, Pittsburgh, PA 15260, USA

**Keywords:** facial expressions, emotions, ambiguity, adolescents, adults, U-shaped trajectory

## Abstract

Understanding the developmental trajectories for recognizing facial expressions is important for a better understanding of development of psychiatric disorders. In this study, we examined the recognition of emotional and neutral facial expressions in 93 typically developing adolescents and adults. The Emotion Intensity Rating task required participants to rate the intensity of emotional expression in happy, neutral, and sad faces on a scale from 1 to 9. A score of ‘5’ had to be assigned to neutral faces, scores between ‘6’ (slightly happy) and ‘9’ (very happy) to happy faces, and scores between ‘4’ (slightly sad) and ‘1’ (very sad) to sad faces. Mixed effects models were used to examine the effects of age and emotion on recognition accuracy, reaction time (RT), and emotional intensity. Participants tended to misjudge neutral faces as sad. Adolescents were less accurate than adults for neutral face recognition. There were significant quadratic effects of age on accuracy (negative quadratic effect) and RT (positive quadratic effect). The most accurate and fastest performance was observed in 25- to 35-year-old subjects. This trajectory may be associated with prefrontal cortex maturation, which provides top–down control over the heightened amygdala response to ambiguity that may be misinterpreted as emotional content.

## 1. Introduction

The recognition of emotional facial expressions is a key aspect of social cognition [1,2] and develops during childhood and adolescence [3,4]. Aberrant recognition of facial emotional expression has been linked to various psychiatric disorders and poor socioemotional functioning [5,6,7,8,9,10,11,12,13]. Therefore, understanding the developmental trajectories for recognizing positive, negative, and neutral facial expressions is important for a better understanding of psychopathological development.

The ability to recognize salient emotions (e.g., happy or sad faces) develops early in life [14,15] and is similar across cultures [16,17] and for adolescents and adults [18], possibly due to attentional biases to the socio-affective cues that emotional faces contain [11]. In contrast, neutral facial expressions are ambiguous [19,20]. It was noted that children and adolescents have difficulty identifying neutral faces as neutral and tend to assign emotional valence to these faces [3,4]. One reason for the non-linear development of the abilities to recognize salient and neutral facial expressions in children and adolescents is that detecting ambiguity relies on top–down processes [21]. These processes support social and emotional cognition [22], rely on amygdala and prefrontal cortex maturation [23,24,25], and develop later in life [21]. 

The goal of this study was to examine the recognition of emotional and ambiguous neutral facial expressions during adolescence and adulthood in typically developing individuals without a history of psychiatric or major systemic disorders. All participants performed the Emotion Intensity Recognition task [26], in which they were asked to judge the emotional intensity of happy, neutral, and sad facial expressions. Participants were informed that there were no correct answers and that judgments had to be made based on the participant’s perception of each face. We found that these instructions make the task more sensitive to individual differences in understanding facial emotional expression because they do not require participants to look for a ground truth regarding emotional categories (e.g., “I think most people would consider this face neutral, so I should respond that it is neutral even though it seems sad to me”). Previous research showed that identifying neutral faces is less accurate, not only in adolescents, but also adults, due to the ambiguous nature of these stimuli [26,27]. Considering that the ability to disentangle ambiguity develops later than the processing of salient stimuli [28,29,30,31], we hypothesize that adolescents will be less accurate than adults in recognizing neutral facial expressions and in recognizing neutral compared to emotional facial expressions [30].

## 2. Materials and Methods

### 2.1. Participants

This study utilized data from two different multimodal neuroimaging studies (R01MH114870 for the adult sample; R01MH114881 and Chuck Noll Foundation for Brain Injury Research FP00004146 for the adolescent sample). Our adult study investigated neural correlates of memory and emotions in individuals with mood disorders compared to healthy controls. Our adolescent study examined brain function in adolescents with concussions compared to healthy non-concussed adolescents. Only healthy controls from the adult and adolescent datasets were included in the current analyses. Both studies were approved by the University of Pittsburgh Institutional Review Board (IRB). The participants were fifty-eight adults between 18 and 45 years old (Mean (SD) age = 28.9 (6.4) years; 43 female; IRB number: STUDY20060265) and thirty-five adolescents between 12 and 17 years of age (Mean (SD) age = 15.3 (1.4) years; 15 female; IRB number: STUDY19030360). All sample sizes were calculated to obtain reliable neuroimaging data according to the specific aims for each study. Written informed consent was obtained from all participants older than 18 years of age. Written informed assent was obtained from all participants younger than 18 years of age, and written informed consent was obtained from their caregivers. All participants were right-handed, fluent in English, and did not have a history of psychiatric disorders based on the age-appropriate psychiatric assessments: SCID-5 [32] for adults and the M.I.N.I.-KID [33] for adolescents. 

### 2.2. Study Procedures

**Figure 1:**
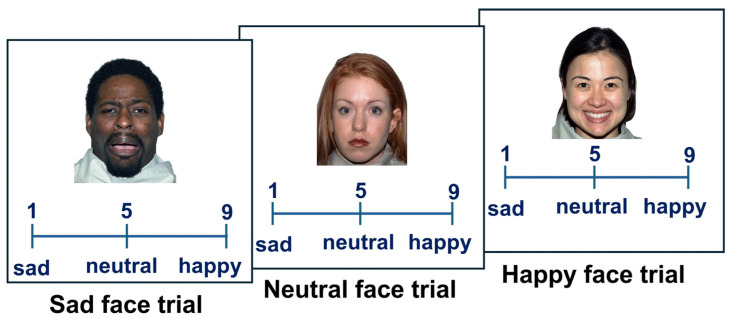
Examples of sad, neutral, and happy face trials in the Emotion Intensity Rating task. The images 40M_SA_O, 01F_NE_C, and 18F_HA_O are taken from the NimStim dataset [36]. The correct responses for the sad facial expression are between ‘1’ and ‘4’. The correct response to the neutral facial expression is ‘5’. The correct response to the happy facial expression is between ‘6’ and ‘9’.

All participants performed the Emotion Intensity Rating task [26], in which they rated the intensity of emotional expression in happy, neutral, and sad faces on a scale from 1 to 9 (Figure 1). The faces and the original emotional categories were taken from the Karolinska Directed Emotional Faces (KDEF) [34,35] and NimStim [36] databases. Adolescents also performed a second task, in which they rated happy, neutral, and angry faces instead of sad faces. The results of the experiment with angry faces are reported in the Supplementary Materials (Figures S1–S3). These faces have previously been tested and are empirically proven to correspond to the indicated emotions. All faces were presented on a white background, were approximately 10–12 degrees of visual angle in size, and were located in the center of the screen. The stimuli were presented in a random order, one at a time, with the rating scale displayed below each face (Figure 1). Participants responded by pressing the corresponding buttons on the PC keyboard. Faces believed to be neutral had to receive a score of ‘5’. Happy faces were scored between ‘6’ (slightly happy faces) and ‘9’ (very happy faces). Sad faces were scored between ‘4’ (slightly sad faces) and ‘1’ (very sad faces). Participants were informed that there were no correct or wrong answers and were asked to judge faces based on their perception of each face. The task was self-paced. Participants were asked to respond as quickly as possible, but could take as much time as they needed. The inter-trial interval was 1000 msec for adults, to reduce fatigue due to a longer task, and 500 msec for adolescents, which was consistent with Experiment 1 in Manelis et al. (2019) [26]. Owing to the differences in research goals for the adult and adolescent studies included in this manuscript, the respective Emotion Intensity Rating tasks each had a different number of trials. In the adult sample, the task included 96 trials, with 32 trials each for sad, happy, and neutral faces. In the adolescent sample, the task included 20 trials, with 10 neutral, 5 happy, and 5 sad face trials. The adolescent sample also performed a similar task with 10 neutral, 5 happy, and 5 angry face trials. The results from the latter experiment are reported in the Supplemental Materials. The outcome variables were emotional expression recognition accuracy, reaction time (RT), and estimated intensity (rating) of emotional expressions. High emotional intensity would correspond to the scores ‘9’ (for happy faces) and ‘1’ (for sad faces), and low intensity would correspond to the scores ‘6’ (for happy faces) and ‘4’ (for sad faces).

### 2.3. Data Analysis

All statistical analyses were conducted using R (https://www.r-project.org (accessed on 15 August 2024)). Mixed effects linear models (‘lme4’ package in R (Bates et al., 2015) [37]) were used to examine the effects of age, sex, and facial emotional expressions on recognition accuracy, RT, and emotional intensity. These models were used to estimate contrasts and means using a ‘modebased’ package in R (Makowski et al., 2020) [38] with Tukey’s Honestly Significant Difference (HSD) correction for multiple comparisons. The participant’s biological sex was added as a covariate. The first analysis tested our hypothesis regarding the differences in the recognition of emotional and neutral facial expressions by adolescents and adults. The second analysis examined the linear and quadratic effects of age on the ability to recognize neutral facial expressions. An additional analysis examined affective bias during the processing of neutral faces by comparing the mean intensity rating for neutral faces in each age group of participants with the score of “5” using a one-sample t-test. Significantly lower mean ratings suggest negative emotional bias, while significantly higher mean ratings suggest positive emotional bias when processing ambiguous neutral faces. The data were plotted using the ‘ggpubr’ and ‘visreg’ libraries in R. 

## 3. Results

**Figure 2:**
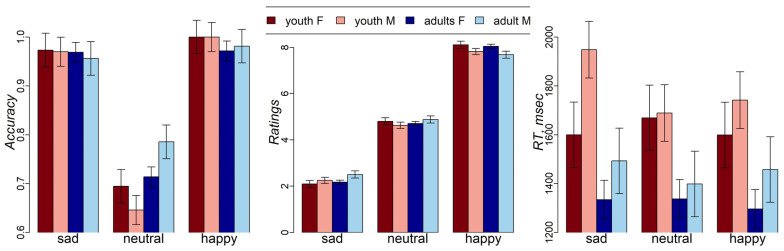
Accuracy, intensity ratings, and RT in adolescent (13–17 yo) and adult (18–45 yo) male and female participants. Standard error bars represent standard errors, estimated from the mixed effects model. F—female, M—male.

The mixed effects analysis of the interactions among emotions, age group, and sex showed a significant emotions-by-age group interaction (F(2,178) = 3.4, *p* = 0.03) and a main effect of emotions (F(2,178) = 106.2, *p* < 0.001) on facial expression recognition accuracy (Figure 2). Lower accuracy was observed for neutral, compared to happy (t(178) = −13.1, *p* < 0.001) and sad (t(178) = −12.1, *p* < 0.001), facial expressions. This effect was more pronounced in adolescents than in adults. There was no effect of sex on recognition accuracy.

As expected, the ratings for happy, neutral, and sad facial expressions significantly differed (F(2,267) = 1750.8, *p* < 0.001) with happy facies having highest ratings (estimated mean (SE) = 7.9 (0.07)), followed by neutral faces (estimated mean (SE) = 4.75 (0.07)), and sad faces (estimated mean (SE) = 2.25 (0.07)). A one-sample t-test revealed that intensity ratings for neutral faces were significantly lower than “5” in both adolescents (t(34) = −4.3, *p* < 0.001) and adults (t(57) = −7.4, *p* < 0.001). Also, there was an interaction between emotions and sex (F(2,267) = 4.5, *p* = 0.01). Females gave higher intensity ratings to happy and sad faces than males (happy mean (SE): female = 8.08 (0.09) vs. male = 7.75 (0.1), sad mean (SE): female = 2.13 (0.09), male = 2.38 (0.1)), but not for neutral faces (mean for both female and male participants = 4.75).

There was a main effect of age group on RT, with adults showing faster performance than adolescents (F(1,89) = 9.7, *p* = 0.002; t = −4.6, *p* < 0.001). We observed no interaction effects on ratings and RT. No effect of sex on RT was observed.

### Quadratic Effect of Age on Recognition of Neutral Facial Expressions

**Figure 3:**
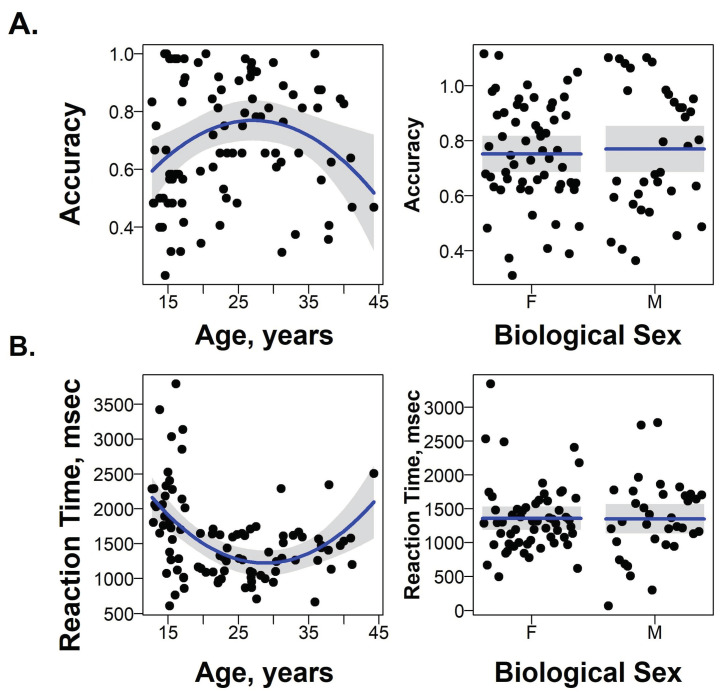
The effects of age and sex on accuracy (**A**) and RT (**B**) for recognizing neutral facial expressions.

A linear regression model included terms for linear and quadratic effects of age and participants’ biological sex. There was a significant negative quadratic effect of age on accuracy (F(1,89) = 6.8, *p* = 0.01; t = −2.6, *p* = 0.01; Figure 3A), and a significant positive quadratic effect of age on RT (F(1,89) = 9.5, *p* = 0.003; t = 3.0, *p* = 0.004; Figure 3B) during the recognition of neutral facial expressions. The highest response accuracy and fastest RTs were observed in participants who were 25–35 years old. Emotion intensity ratings for neutral faces did not depend on either linear or quadratic effects of participant’s age. No effects of sex on recognition accuracy, ratings, or RT were observed.

## 4. Discussion

This study examined the recognition of emotional facial expressions in healthy adolescents and young adults. Participants accurately recognized happy and sad facial expressions independently of their age. Consistent with previous findings [26,27,30], the accuracy for neutral facial expressions was significantly lower than those for happy and sad facial expressions across all participants. Both adolescents and adults tended to misjudge neutral faces as sad, thus showing negative affective bias. Overall, adolescents had lower accuracy and slower RTs when recognizing neutral facial expressions than adults. Interestingly, the relationship between participants’ age and accuracy followed an inverted U-shaped curve, while the relationship between participants’ age and RT followed a U-shaped curve.

Neutral facial expressions are ambiguous [19,20]. Ambiguous stimulus processing requires sufficient cognitive resources [39] to exhibit top–down control to orient social attention [21]. Previous studies suggested that the development of cognitive control follows an inverted U-shaped trajectory [40,41], which may be related to an inverted U-shaped curve in the development of the frontoparietal control network, which reaches its peak between 24 and 41 years of age [40]. Our findings are consistent with this notion and demonstrate that the youngest and oldest participants show less accurate and slower performance, while those between 25 and 35 years of age show the most accurate and fastest recognition of neutral faces. 

One explanation for an inverted U-shaped developmental trajectory for neutral face recognition is that adolescents do not have sufficient resources to apply top–down cognitive control to rule out emotional signals due to underdeveloped emotional differentiation [29]. On the other hand, adolescents may have difficulty with recognizing neutral facial expressions due to increased sensitivity to emotions and social rejection [42], rather than a reduced ability to process ambiguous stimuli. The latter idea is consistent with the findings of an inverted U-shaped developmental trajectory and overall heightened amygdala activation in response to emotions during adolescence [23,43]. Considering that the amygdala is engaged in the coding of categorical ambiguity [44] and that amygdala activation increases can lead to interpreting neutral stimuli as emotional, the development of the ability to tell apart emotional and neutral stimuli might follow the developmental trajectory of the amygdala. 

Understanding other people’s facial expressions is related to social cognition and empathy and is necessary for appropriate behavioral responses in a social environment [1,2]. Aberrant recognition of emotional facial expressions and reduced empathy were linked to poor social skills [45] and more severe symptoms of substance use [46,47], autism spectrum disorder [24], and depressive [26,48] disorders. Considering that various psychiatric disorders, including depressive and substance use disorders, develop during adolescence [49,50], it is important to understand how ambiguity development is related to the development of psychiatric disorders in adolescents and young adults. For example, interpreting neutral facial expressions as emotional may increase anxiety, thus leading to inappropriate emotional or behavioral responses. Previous studies showed that anxious children tend to anticipate negative emotions in ambiguous situations [28]. 

Several previous studies reported the effect of participants’ sex on emotional processing [51,52,53]. Our study showed that female participants, compared to male participants, gave higher intensity ratings to happy and sad faces. However, there were no differences between females and males in intensity ratings for neutral faces, nor in response speed and accuracy for all emotions. These findings suggest that females experience a more intense emotional response not only to negative [54], but also to positive, emotional stimuli. Future studies should investigate further whether such factors as emotional intelligence, empathy, or other internal traits or external factors differentially increase the perceived intensity of facial emotional expressions in males and females. 

Although we discuss here the potential neurobiological underpinnings of the ambiguity processing development, our study did not collect functional neuroimaging data during the Emotion Intensity recognition task. Future studies should address this limitation. Another possible limitation is the relative age of the facial stimuli. Given that all faces belonged to adults, it is unclear how the ability to disentangle neutral from emotional faces in adolescents would change if the facial stimuli were age matched. In addition, the upper age range of our participants was limited to 45 years. Future research should examine the processing of ambiguous emotional stimuli in older adults, especially in the relationship to developing late-life depression. 

In summary, our study revealed that the ability to recognize ambiguous neutral facial expressions develops during adolescence and early adulthood and follows an inverted U-shaped trajectory, with the peak occurring between 25 and 35 years of age. This developmental trajectory may be associated with the maturation of the prefrontal cortex, which provides top–down control over the heightened amygdala response to stimulus ambiguity that may be misinterpreted as emotional content. Further work is needed to understand the relationship between the ability to interpret neutral facial expressions and the development of psychiatric disorders across the lifespan.

## Data Availability

The data presented in this study are available on request from the corresponding author.

## Supplementary Materials

The following supporting information can be downloaded at: https://www.mdpi.com/article/10.3390/bs14090826/s1, Figure S1: Accuracy, ratings, and RT for the Emotion Intensity Rating tasks with sad and angry stimuli in adolescents; Figure S2: Estimated means for accuracy, intensity ratings, and RT in youth (13–17 yo) and adult (18–45 yo) samples. Standard error bars represent standard errors estimated from the mixed effects model; Figure S3: The effect of age on accuracy (A) and RT (B) for recognizing neutral facial expressions.

## References

[1] Moret-Tatay C., Mundi-Ricós P., Irigaray T.Q. (2023). The Relationship between Face Processing, Cognitive and Affective Empathy. Behav. Sci..

[2] Choi D., Watanuki S. (2014). Effect of Empathy Trait on Attention to Faces: An Event-Related Potential (ERP) Study. J. Physiol. Anthropol..

[3] Durand K., Gallay M., Seigneuric A., Robichon F., Baudouin J.Y. (2007). The Development of Facial Emotion Recognition: The Role of Configural Information. J. Exp. Child Psychol..

[4] Pfaltz M.C., Passardi S., Auschra B., Fares-Otero N.E., Schnyder U., Peyk P. (2019). Are You Angry at Me? Negative Interpretations of Neutral Facial Expressions Are Linked to Child Maltreatment but Not to Posttraumatic Stress Disorder. Eur. J. Psychotraumatol..

[5] Foisy M.L., Kornreich C., Petiau C., Parez A., Hanak C., Verbanck P., Pelc I., Philippot P. (2007). Impaired Emotional Facial Expression Recognition in Alcoholics: Are These Deficits Specific to Emotional Cues?. Psychiatry Res..

[6] Brotman M.A., Guyer A.E., Lawson E.S., Horsey S.E., Rich B.A., Dickstein D.P., Pine D.S., Leibenluft E. (2008). Facial Emotion Labeling Deficits in Children and Adolescents at Risk for Bipolar Disorder. Am. J. Psychiatry.

[7] Leppänen J.M. (2006). Emotional Information Processing in Mood Disorders: A Review of Behavioral and Neuroimaging Findings. Curr. Opin. Psychiatry.

[8] Freeman C.R., Wiers C.E., Sloan M.E., Zehra A., Ramirez V., Wang G.J., Volkow N.D. (2018). Emotion Recognition Biases in Alcohol Use Disorder. Alcohol Clin. Exp. Res..

[9] Cservenka A., Donahue L.C. (2024). Emotion Recognition and Self-Reported Emotion Processing in Alcohol and Cannabis Co-Using Young Adults. Behav. Sci..

[10] Gao Z., Zhao W., Liu S., Liu Z., Yang C., Xu Y. (2021). Facial Emotion Recognition in Schizophrenia. Front. Psychiatry.

[11] Pabst A., Bollen Z., Masson N., Billaux P., de Timary P., Maurage P. (2023). An Eye-Tracking Study of Biased Attentional Processing of Emotional Faces in Severe Alcohol Use Disorder. J. Affect. Disord..

[12] Staff A.I., Luman M., van der Oord S., Bergwerff C.E., van den Hoofdakker B.J., Oosterlaan J. (2022). Facial Emotion Recognition Impairment Predicts Social and Emotional Problems in Children with (Subthreshold) ADHD. Eur. Child Adolesc. Psychiatry.

[13] Szanto K., Dombrovski A.Y., Sahakian B.J., Mulsant B.H., Houck P.R., Reynolds C.F., Clark L. (2012). Social Emotion Recognition, Social Functioning, and Attempted Suicide in Late-Life Depression. Am. J. Geriatr. Psychiatry.

[14] Leppänen J.M., Nelson C.A. (2009). Tuning the Developing Brain to Social Signals of Emotions. Nat. Rev. Neurosci..

[15] Lawrence K., Campbell R., Skuse D. (2015). Age, Gender, and Puberty Influence the Development of Facial Emotion Recognition. Front. Psychol..

[16] Ekman P., Friesen W.V. (1971). Constants across Cultures in the Face and Emotion. J. Pers. Soc. Psychol..

[17] Ekman P., Friesen W.V., O’Sullivan M., Chan A., Diacoyanni-Tarlatzis I., Heider K., Krause R., LeCompte W.A., Pitcairn T., Ricci-Bitti P.E. (1987). Universals and Cultural Differences in the Judgments of Facial Expressions of Emotion. J. Pers. Soc. Psychol..

[18] Rodger H., Sokhn N., Lao J., Liu Y., Caldara R. (2023). Developmental Eye Movement Strategies for Decoding Facial Expressions of Emotion. J. Exp. Child Psychol..

[19] Carvajal F., Rubio S., Serrano J.M., Ríos-Lago M., Alvarez-Linera J., Pacheco L., Martín P. (2013). Is a Neutral Expression Also a Neutral Stimulus? A Study with Functional Magnetic Resonance. Exp. Brain Res..

[20] Rollins L., Bertero E., Hunter L. (2021). Developmental Differences in the Visual Processing of Emotionally Ambiguous Neutral Faces Based on Perceived Valence. PLoS ONE.

[21] Kaminska O.K., Magnuski M., Olszanowski M., Gola M., Brzezicka A., Winkielman P. (2020). Ambiguous at the Second Sight: Mixed Facial Expressions Trigger Late Electrophysiological Responses Linked to Lower Social Impressions. Cogn. Affect. Behav. Neurosci..

[22] Vijayakumar N., Pfeifer J.H., Flournoy J.C., Hernandez L.M., Dapretto M. (2019). Affective Reactivity during Adolescence: Associations with Age, Puberty and Testosterone. Cortex.

[23] Moore W.E., Pfeifer J.H., Masten C.L., Mazziotta J.C., Iacoboni M., Dapretto M. (2012). Facing Puberty: Associations between Pubertal Development and Neural Responses to Affective Facial Displays. Soc. Cogn. Affect. Neurosci..

[24] O’Hearn K., Lynn A. (2023). Age Differences and Brain Maturation Provide Insight into Heterogeneous Results in Autism Spectrum Disorder. Front. Hum. Neurosci..

[25] Scherf K.S., Smyth J.M., Delgado M.R. (2013). The Amygdala: An Agent of Change in Adolescent Neural Networks. Horm. Behav..

[26] Manelis A., Huppert T.J., Rodgers E., Swartz H.A., Phillips M.L. (2019). The Role of the Right Prefrontal Cortex in Recognition of Facial Emotional Expressions in Depressed Individuals: FNIRS Study. J. Affect. Disord..

[27] Somerville L.H., Kim H., Johnstone T., Alexander A.L., Whalen P.J. (2004). Human Amygdala Responses during Presentation of Happy and Neutral Faces: Correlations with State Anxiety. Biol. Psychiatry.

[28] Dodd H.F., Stuijfzand S., Morris T., Hudson J.L. (2015). Child Anxiety and the Processing of Ambiguity. Cognit. Ther. Res..

[29] Nook E.C., Sasse S.F., Lambert H.K., McLaughlin K.A., Somerville L.H. (2018). The Nonlinear Development of Emotion Differentiation: Granular Emotional Experience Is Low in Adolescence. Psychol. Sci..

[30] Wiggins J.L., Adleman N.E., Kim P., Oakes A.H., Hsu D., Reynolds R.C., Chen G., Pine D.S., Brotman M.A., Leibenluft E. (2015). Developmental Differences in the Neural Mechanisms of Facial Emotion Labeling. Soc. Cogn. Affect. Neurosci..

[31] Lee T.H., Perino M.T., McElwain N.L., Telzer E.H. (2020). Perceiving Facial Affective Ambiguity: A Behavioral and Neural Comparison of Adolescents and Adults. Emotion.

[32] Structured Clinical Interview for DSM-5—Research Version (SCID-5 for DSM-5, Research Version; SCID-5-RV). https://www.appi.org/getattachment/7c0e352e-eff3-4f55-9266-86454db15cd6/1-SCID-5-RV_Score_sheet_V1-0-0.pdf.

[33] Sheehan D.V., Sheehan K.H., Shytle R.D., Janavs J., Bannon Y., Rogers J.E., Milo K.M., Stock S.L., Wilkinson B. (2010). Reliability and Validity of the Mini International Neuropsychiatric Interview for Children and Adolescents (MINI-KID). J. Clin. Psychiatry.

[34] Garrido M.V., Prada M. (2017). KDEF-PT: Valence, Emotional Intensity, Familiarity and Attractiveness Ratings of Angry, Neutral, and Happy Faces. Front. Psychol..

[35] Goeleven E., De Raedt R., Leyman L., Verschuere B. (2008). The Karolinska Directed Emotional Faces: A Validation Study. Cogn. Emot..

[36] Tottenham N., Tanaka J.W., Leon A.C., McCarry T., Nurse M., Hare T.A., Marcus D.J., Westerlund A., Casey B.J., Nelson C. (2009). The NimStim Set of Facial Expressions: Judgments from Untrained Research Participants. Psychiatry Res..

[37] Bates D., Mächler M., Bolker B.M., Walker S.C. (2015). Fitting Linear Mixed-Effects Models Using lme4. J. Stat. Softw..

[38] Makowski D., Ben-Shachar M.S., Patil I., Lüdecke D. (2020). Methods and Algorithms for Correlation Analysis in R. J. Open Source Softw..

[39] Abubshait A., Momen A., Wiese E. (2020). Pre-Exposure to Ambiguous Faces Modulates Top-Down Control of Attentional Orienting to Counterpredictive Gaze Cues. Front. Psychol..

[40] Li Z., Petersen I.T., Wang L., Radua J., Yang G., Liu X. (2024). The Lifespan Trajectories of Brain Activities Related to Cognitive Control. bioRxiv.

[41] Erb C.D., Germine L., Hartshorne J.K. (2023). Cognitive Control Across the Lifespan: Congruency Effects Reveal Divergent Developmental Trajectories. J. Exp. Psychol. Gen..

[42] Sebastian C.L. (2015). Social Cognition in Adolescence: Social Rejection and Theory of Mind. Psicol. Educ..

[43] Pagliaccio D., Luby J.L., Gaffrey M.S., Belden A.C., Botteron K.N., Harms M.P., Barch D.M. (2013). Functional Brain Activation to Emotional and Nonemotional Faces in Healthy Children: Evidence for Developmentally Undifferentiated Amygdala Function during the School-Age Period. Cogn. Affect. Behav. Neurosci..

[44] Wang S., Yu R., Tyszka J.M., Zhen S., Kovach C., Sun S., Huang Y., Hurlemann R., Ross I.B., Chung J.M. (2017). The Human Amygdala Parametrically Encodes the Intensity of Specific Facial Emotions and Their Categorical Ambiguity. Nat. Commun..

[45] D’Hondt F., Campanella S., Kornreich C., Philippot P., Maurage P. (2014). Below and beyond the Recognition of Emotional Facial Expressions in Alcohol Dependence: From Basic Perception to Social Cognition. Neuropsychiatr. Dis. Treat..

[46] Rupp C.I., Derntl B., Osthaus F., Kemmler G., Fleischhacker W.W. (2017). Impact of Social Cognition on Alcohol Dependence Treatment Outcome: Poorer Facial Emotion Recognition Predicts Relapse/Dropout. Alcohol Clin. Exp. Res..

[47] Valmas M.M., Mosher Ruiz S., Gansler D.A., Sawyer K.S., Oscar-Berman M. (2014). Social Cognition Deficits and Associations with Drinking History in Alcoholic Men and Women. Alcohol Clin. Exp. Res..

[48] Guhn A., Merkel L., Hübner L., Dziobek I., Sterzer P., Köhler S. (2020). Understanding versus Feeling the Emotions of Others: How Persistent and Recurrent Depression Affect Empathy. J. Psychiatr. Res..

[49] Paus T., Keshavan M., Giedd J.N. (2008). Why Do Many Psychiatric Disorders Emerge during Adolescence?. Nat. Rev. Neurosci..

[50] Ahmed S.P., Bittencourt-Hewitt A., Sebastian C.L. (2015). Neurocognitive Bases of Emotion Regulation Development in Adolescence. Dev. Cogn. Neurosci..

[51] Li H., Yuan J., Lin C. (2008). The Neural Mechanism Underlying the Female Advantage in Identifying Negative Emotions: An Event-Related Potential Study. Neuroimage.

[52] Yuan J., Luo Y., Yan J.H., Meng X., Yu F., Li H. (2009). Neural Correlates of the Females’ Susceptibility to Negative Emotions: An Insight into Gender-Related Prevalence of Affective Disturbances. Hum. Brain Mapp..

[53] Kret M.E., De Gelder B. (2012). A Review on Sex Differences in Processing Emotional Signals. Neuropsychologia.

[54] Gard M.G., Kring A.M. (2007). Sex Differences in the Time Course of Emotion. Emotion.

