# Supplementary material for: The Development of Ambiguity Processing Is Explained by an Inverted U-Shaped Curve
Source: Behav Sci (Basel). 2024 Sep 16;14(9):826. doi: 10.3390/bs14090826 (PMC11429131; doi:10.3390/bs14090826)
Supplement: Supplementary file 1 [file behavsci-14-00826-s001.zip › behavsci-3138776-supplementary.pdf]

SUPPLEMENTARY MATERIALS

The effect of emotions on accuracy, rating, and RT in the two Emotion Intensity Rating tasks in adolescents.

Adolescents performed two Emotion Intensity Rating task [26]. In the first task, they rated the intensity of facial emotional expression in happy, neutral, and sad faces. In the second task, they rated the intensity of facial emotional expression in happy, neutral, and angry faces. All ratings were on a scale of 1 to 9. The faces and the original emotion categories were taken from the Karolinska Directed Emotional Faces (KDEF) [34,35] and NimStim [36] databases.

Statistical analyses were conducted using R (https://www.r-project.org). Mixed effects linear models ('lme4' package in R (Bates et al., 2015) [37]) were used to examine the effects of facial emotional expressions on recognition accuracy, emotion intensity ratings, and RT. These models were used to estimate contrasts and means using a 'modebased' package in R (Makowski et al., 2020) [38] with Tukey's Honestly Significant Difference (HSD) correction for multiple comparisons.

There was a significant effect of emotions on accuracy (task with sad faces: $F(2,102)=54.5$, $p<0.001$; task with angry faces: $F(2,102)=62.5$, $p<0.001$), ratings (task with sad faces: $F(2,68)=713$, $p<0.001$; task with angry faces: $F(2,68)=862$, $p<0.001$), but not RT ($p>0.1$). **Figure S1** illustrates the results. Neutral faces were recognized less accurately than happy (task with sad faces: $t(68)= -9.4$, $p<0.001$; task with angry faces: $t(68)= -9.8$, $p<0.001$), sad ($t(68)= -8.6$, $p<0.001$), and angry faces ($t(68)= -9.5$, $p<0.001$).

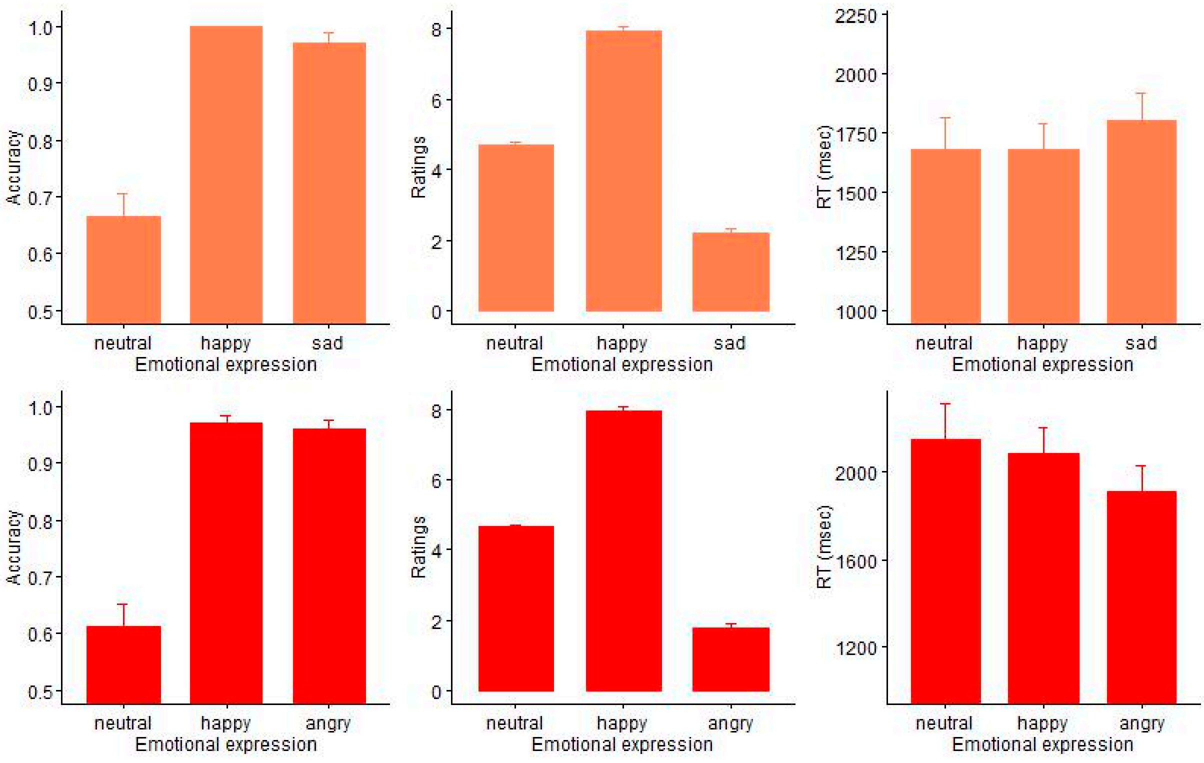

Figure S1. Accuracy, ratings, and RT for the Emotion Intensity Rating tasks with sad and angry stimuli in adolescents.

Considering that the number of trials in the adolescent sample was low (10 neutral faces), we decided to aggregate neutral and happy faces across the two tasks described above in adolescents to obtain better estimates for these conditions. We then combined adolescent and adult data and re-analyzed the emotion-by-age group interaction and quadratic effect of age models

The mixed effects analysis with emotion-by-age group interaction and sex as a covariate showed a significant emotion-by-age group interaction effect (F(2,272)=4.66, p=0.01) and a main effect of emotions (F(2,272)=149.2, p<0.001) on facial expressions recognition accuracy (**Figure S2**). Lower accuracy was observed for neutral, compared to happy (t(182)=-15.25, p<0.001) and sad (t(182)=-14.66, p<0.001), facial expressions. This effect was more pronounced in youth than in adults (t=-3.46, p=0.008). No effect of sex on recognition accuracy was observed.

As expected, the ratings for happy, neutral, and sad facial expressions significantly differed (F(2,272)=2104.21, p<0.001) with happy facies having highest ratings (estimated mean(SE)=7.95(0.06)), followed by neutral faces (estimated mean(SE)=4.71(0.06)), and sad faces (estimated mean(SE)=2.22(0.06)). A one-sample t-test revealed that intensity ratings for neutral faces were significantly lower than "5" in both youth (t(34)= -5.4, p<0.001) and adults (t(57)= -7.4, p<0.001) groups. No effect of sex on the ratings was observed.

There was a main effect of the age group on RT with adults showing faster performance than youth (F(1,90)=23.6, p<0.001; t= -4.6, p<0.001). We observed no interaction effects on ratings and RT. No effect of sex on RT was observed.

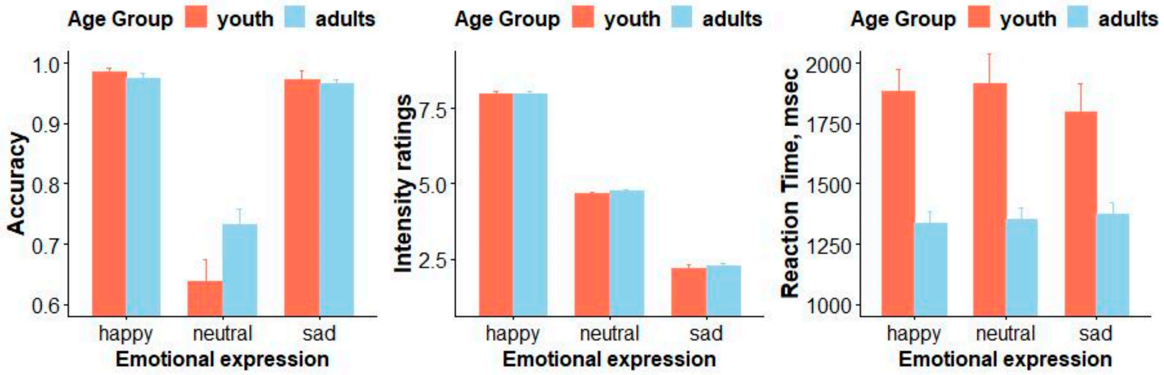

Figure S2. Estimated means for accuracy, intensity ratings, and RT in youth (13-17yo) and adult (18-45yo) samples. Standard error bars represent standard errors estimated from the mixed effects model.

Quadratic effect of age on recognition of neutral facial expressions

A linear regression model included terms for linear and quadratic effects of age and participant's biological sex. There was a significant negative quadratic effect of age on accuracy (F(1,90)=10.8, p<0.005; t= -3.3, p<0.005; **Figure S3A**), and a significant linear F(1,90)=9.2, p<0.005) and positive quadratic F(1,90)=19.4, p<0.001; t=4.4, p<0.001**; Figure S3B**) effects of age on RT during recognition of neutral facial expressions. The highest response accuracy and fastest RT were observed in participants who were 25-35 years old. Emotional intensity ratings for neutral faces did not depend on either linear or quadratic effects of participant's age. No effect of sex on recognition accuracy, ratings, or RT was observed.

The results obtained using the aggregated sample for youth were similar to those we reported in the main paper.

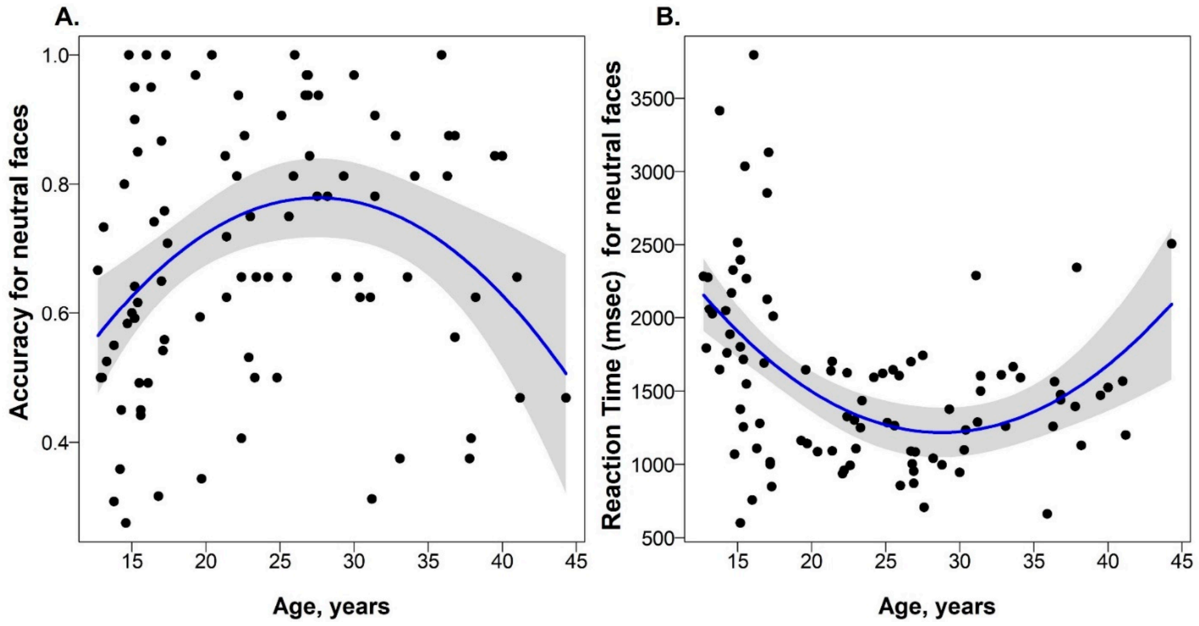

Figure S3. The effect of age on accuracy (A) and RT (B) for recognizing neutral facial expressions.
